# Supplementary figures and images for: The Slit‐binding Ig1 domain is required for multiple axon guidance activities of Drosophila Robo2
Source: Genesis. 2021 Aug 19;59(9):e23443. doi: 10.1002/dvg.23443 (PMC8446337; doi:10.1002/dvg.23443)

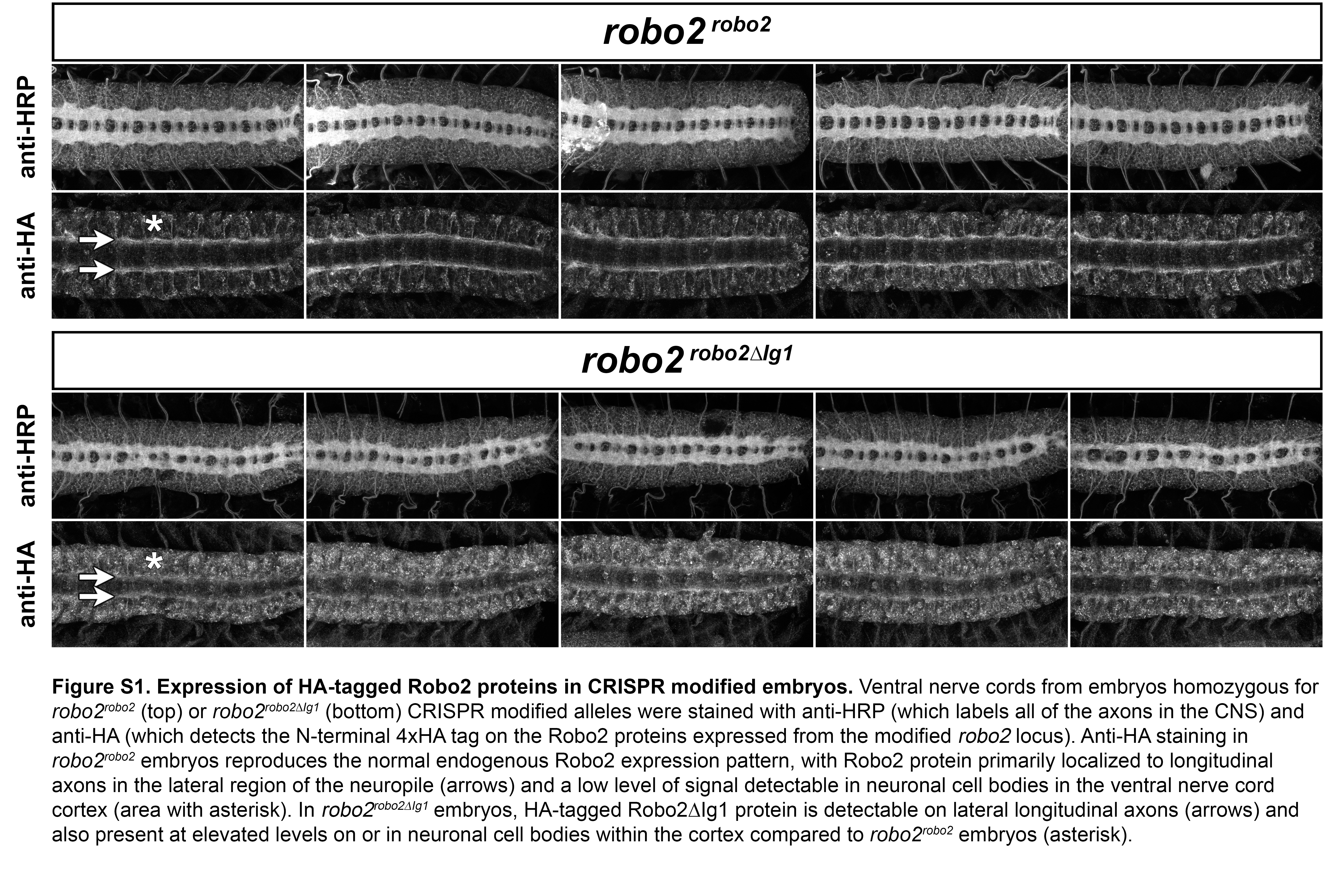

Supplement: Supplementary file 1 — FIGURE S1 Expression of HA‐tagged Robo2 proteins in CRISPR‐modified embryos. Ventral nerve cords (VNCs) from embryos homozygous for robo2 robo2 (top) or robo2 robo2∆Ig1 (bottom) CRISPR‐modified alleles were stained with anti‐HRP (which labels all of the axons in the CNS) and anti‐HA (which detects the N‐terminal 4xHA tag on the Robo2 proteins expressed from the modified robo2 locus). Anti‐HA staining in robo2 robo2 embryos reproduces the normal endogenous Robo2 expression pattern, with Robo2 protein primarily localized to longitudinal axons in the lateral region of the neuropile (arrows) and a low level of signal detectable in neuronal cell bodies in the VNC cortex (area with asterisk). In robo2 robo2∆Ig1 embryos, HA‐tagged Robo2∆Ig1 protein is detectable on lateral longitudinal axons (arrows) and also present at elevated levels on or in neuronal cell bodies within the cortex compared to robo2 robo2 embryos (asterisk). [file DVG-59-e23443-s002.tif]

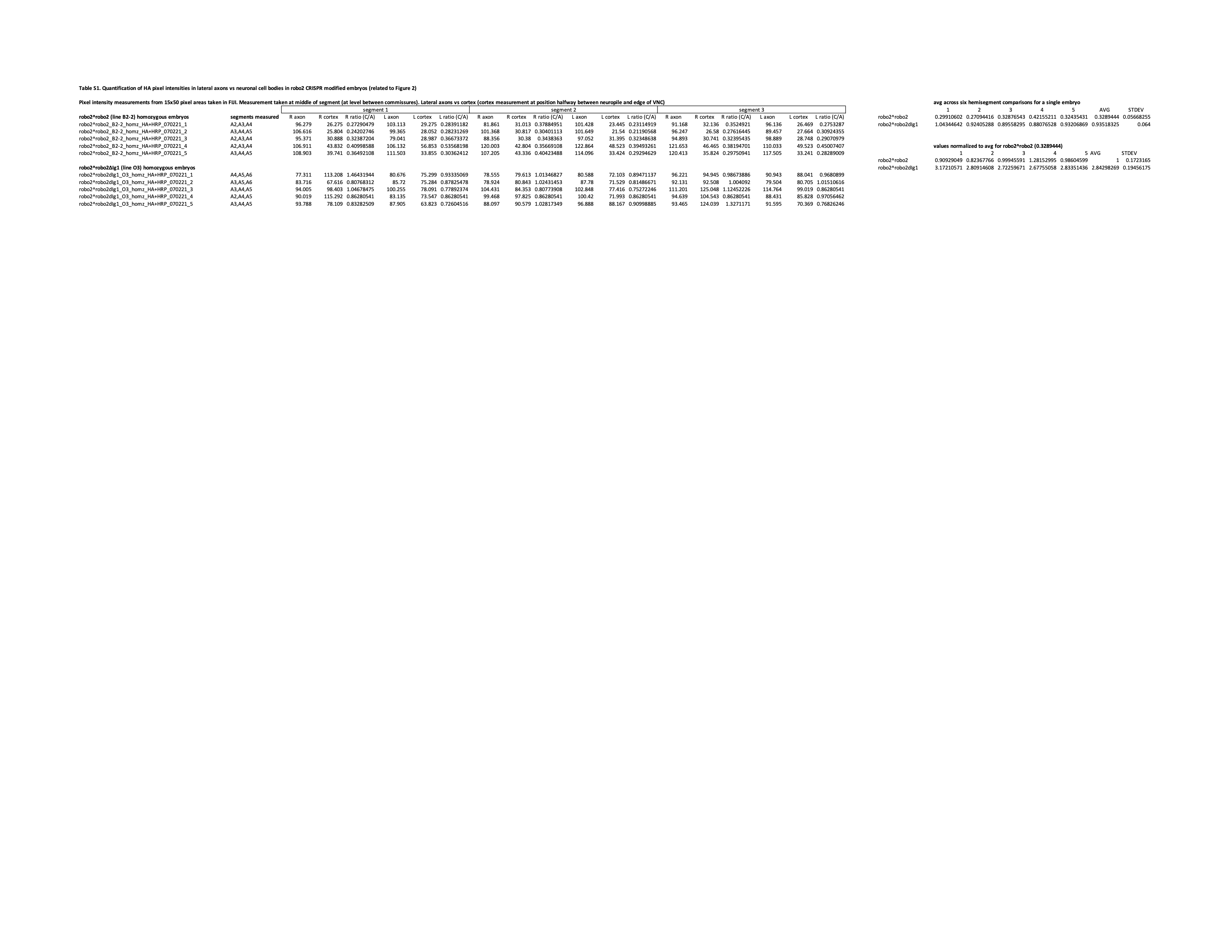

Supplement: Supplementary file 2 — TABLE S1 Supplementary Table [file DVG-59-e23443-s003.tif]

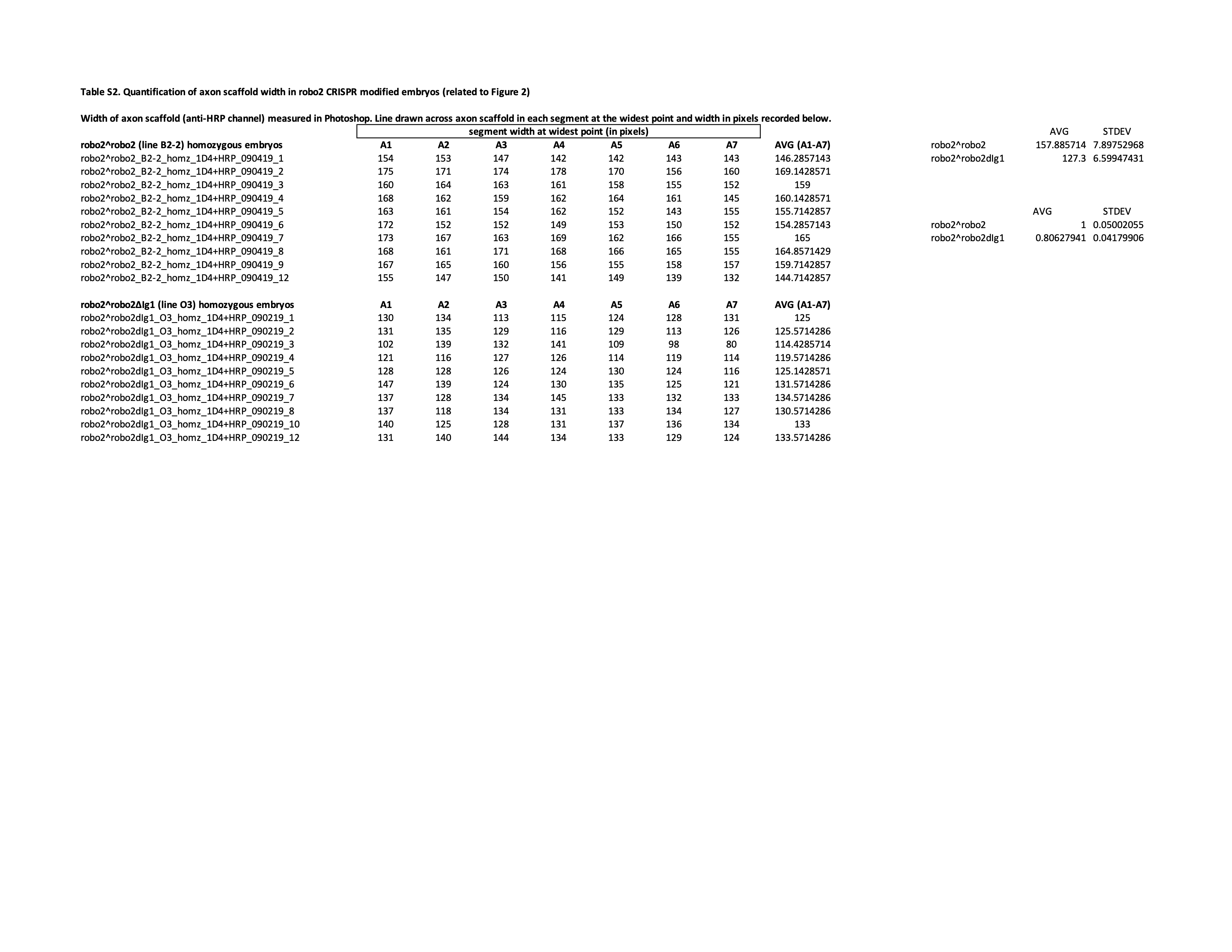

Supplement: Supplementary file 3 — TABLE S2 Supplementary Table [file DVG-59-e23443-s004.tif]

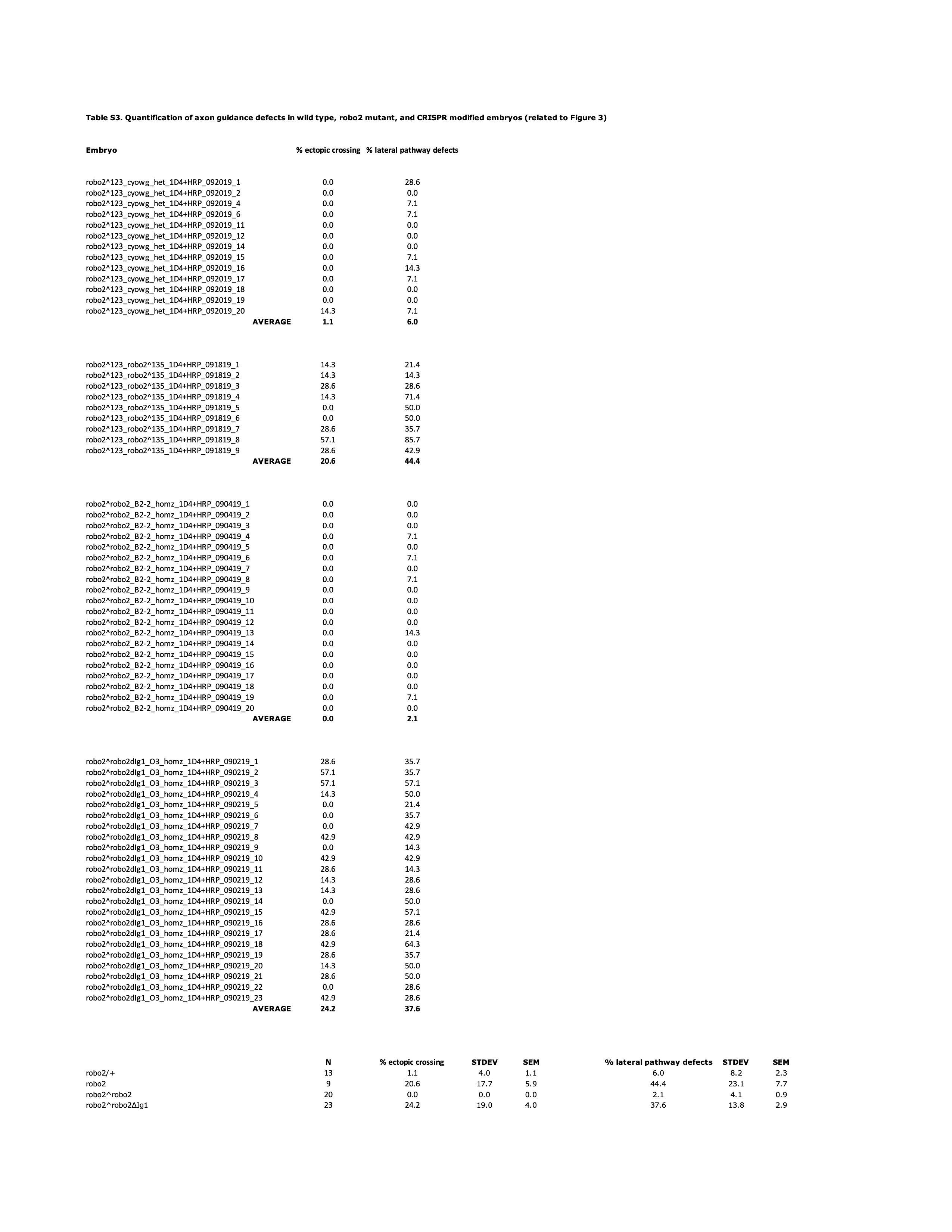

Supplement: Supplementary file 4 — TABLE S3 Supplementary Table [file DVG-59-e23443-s001.tif]
